# Annotated draft genome sequences of three species of *Cryptosporidium*: *Cryptosporidium meleagridis* isolate UKMEL1, *C. baileyi* isolate TAMU-09Q1 and *C. hominis* isolates TU502_2012 and UKH1

**DOI:** 10.1093/femspd/ftw080

**Published:** 2016-08-12

**Authors:** Olukemi O. Ifeonu, Marcus C. Chibucos, Joshua Orvis, Qi Su, Kristin Elwin, Fengguang Guo, Haili Zhang, Lihua Xiao, Mingfei Sun, Rachel M. Chalmers, Claire M. Fraser, Guan Zhu, Jessica C. Kissinger, Giovanni Widmer, Joana C. Silva

**Affiliations:** 1 Institute for Genome Sciences, University of Maryland School of Medicine, Baltimore, MD 21201, USA; 2 *Cryptosporidium* Reference Unit, Public Health Wales Microbiology, Singleton Hospital, Swansea SA2 8QA, UK; 3 Department of Veterinary Pathobiology, Texas A&M University, College Station, TX 77843, USA; 4 Division of Foodborne, Waterborne and Environmental Diseases, National Center for Emerging and Zoonotic Infectious Diseases, Centers for Disease Control and Prevention, Atlanta, GA 30329, USA; 5 Institute of Animal Health, Guangdong Academy of Agricultural Sciences, Guangzhou, Guangdong, China 510640; 6 Center for Tropical and Emerging Global Diseases, Institute of Bioinformatics and Department of Genetics, University of Georgia, Athens, GA 30602, USA; 7 Department of Infectious Disease and Global Health, Tufts University Cummings School of Veterinary Medicine, North Grafton, MA 01536, USA; 8 Department of Microbiology and Immunology, University of Maryland School of Medicine, Baltimore, MD 21201, USA

**Keywords:** *Cryptosporidium*, *C. hominis* TU502_2012, *Cryptosporidium meleagridis*, *Cryptosporidium baileyi*, genome assembly, annotation

## Abstract

Human cryptosporidiosis is caused primarily by *Cryptosporidium hominis*, *C. parvum* and *C. meleagridis.* To accelerate research on parasites in the genus *Cryptosporidium*, we generated annotated, draft genome sequences of human *C. hominis* isolates TU502_2012 and UKH1, *C. meleagridis* UKMEL1, also isolated from a human patient, and the avian parasite *C. baileyi* TAMU-09Q1. The annotation of the genome sequences relied in part on RNAseq data generated from the oocyst stage of both *C. hominis* and *C. baileyi*. The genome assembly of *C. hominis* is significantly more complete and less fragmented than that available previously, which enabled the generation of a much-improved gene set for this species, with an increase in average gene length of 500 bp relative to the protein-encoding genes in the 2004 *C. hominis* annotation. Our results reveal that the genomes of *C. hominis* and *C. parvum* are very similar in both gene density and average gene length. These data should prove a valuable resource for the *Cryptosporidium* research community.

## Abstract (precis)

The release of the draft genome sequence, and corresponding annotation, of *Cryptosporidium baileyi, C. hominis* isolates TU502_2012 and UKH1, and *C. meleagridis*, will accelerate research on *Cryptosporidium* parasites.

*Cryptosporidium* parasites (Phylum: Apicomplexa) infect a wide range of vertebrates, from fish to humans, and are the causative agents of cryptosporidiosis in humans (Upton and Current 1985; Tzipori 1988; Widmer and Sullivan 2012). A recent, large, multicenter study of the etiology of moderate-to-severe diarrhea (MSD) in infants in the developing world found *Cryptosporidium hominis* to be among the four predominant pathogens associated with MSD in children under 5 years of age (Kotloff *et al.*^2013^). Some *Cryptosporidium* species are capable of zoonotic transmission (Ryan, Fayer and Xiao 2014). Comparative analysis of genomes from diverse *Cryptosporidium* species and related protists is essential to fully understand the biology, pathology, host specificity and evolution of this genus.

The reference *C. parvum* IOWA II genome (Abrahamsen *et al.*^2004^) is essentially complete, with its eight chromosomes distributed among 18 contigs, including full-length chromosomes. In contrast, the reference assembly of *C. hominis*, based on isolate TU502, published in 2004 (Xu *et al.*^2004^), is a highly fragmented draft genome consisting of 1422 contigs. To accelerate research on these pathogens of public health and veterinary significance, we sequenced, assembled and annotated four *Cryptosporidium* genome sequences belonging to three species as part of a community White Paper undertaking. Two sequences were generated from a species infective to humans, *C. hominis* isolates TU502_2012 and UKH1. In addition, sequences were generated from the generalist species *C. meleagridis*, isolate UKMEL1, and from the TAMU-09Q1 isolate of *C. baileyi*, an avian-infecting parasite. All three species are enteric parasites. *Cryptosporidium baileyi* can complete its entire life cycle in embryonated chicken eggs, making it a useful laboratory model to address some aspects of *Cryptosporidium* biology. *Cryptosporidium meleagridis* appears to lack host specificity, as it is known to infect both avian and mammalian species (Akiyoshi *et al.*^2003^).

*Cryptosporidium hominis* UKH1 and *C. meleagridis* UKMEL1 oocysts were isolated from fecal samples of naturally infected humans. *Cryptosporidium meleagridis* oocysts were propagated in immunosuppressed adult CD-1 mice, and *C. hominis* UKH1 in neonatal gnotobiotic pigs. *Cryptosporidium hominis* TU502_2012 originates from *C. hominis* TU502 isolate maintained by serial propagation in gnotobiotic pigs (Tzipori *et al.*^1994^; Xu *et al.*^2004^). *Cryptosporidium baileyi* oocysts were extracted from experimentally infected embryonated chicken eggs. Prior to isolating DNA, extracted oocysts were purified on density gradients (Widmer, Feng and Tanriverdi 2004) and surface-sterilized with bleach to minimize contamination with host and bacterial DNA. RNA samples were obtained from *C. hominis* TU502_2012 and *C. baileyi* TAMU-10GZ1 oocysts <4 months old, and sequenced to high coverage using strand-specific RNASeq (Parkhomchuk *et al.*^2009^). *De novo* assembly of the genomic reads was performed using MaSuRCA version1.9 (Zimin *et al.*^2013^) (Table 1).

**Table 1.** Summary statistics of whole-genome sequence and transcriptome data, assemblies and annotation.

	*Cryptosporidium hominis*			*Cryptosporidium*	*Cryptosporidium*
				*meleagridis*	*baileyi*
Isolate: DNA	TU502^*a*^	TU502_2012	UKH1	UKMEL1	TAMU-09Q1
gDNA Illumina library fragment size (bp)	N/A	460	461	517	654
No. MiSeq reads	N/A	6,871,858	7,596,410	22,862,044	6,240,960
No. base pairs	N/A	1,724,836,358	1,906,698,910	6,881,475,244	1,566,480,960
Assembly size (bp)	8,743,570	9,107,739	9,156,091	8,973,200	8,493,640
No. of contigs	1422	119	156	57	145
Contig N_50_	14,504	238,509	179,408	322,908	203,018
Largest contig (bp)	90,444	1,270,815	542,781	732,862	702,637
G + C content (%)	30.9	30.1	30.1	31.0	24.3
No. protein-coding genes	3,885	3,745	3,765	3,758	3,692
Average gene length (bp)	1,360	1,892	1,830	1,844	1,778
Percent coding	60.4%	77.8%	75.2%	77.2%	77.3%
Accession no.	AAEL00000000	JIBM00000000	JIBN00000000	JIBK00000000	JIBL00000000
SNPs relative to TU502^*a*^ synonymous : non-syn		1303 : 2,567	718 : 1336	N/A	N/A
SNPs relative to TU502_2012 synonymous : non-syn		N/A	143 : 339	N/A	N/A
Isolate: RNA		TU502_2012	UKH1	UKMEL1	TAMU-10GZ1
No. HiSeq read pairs		16,568,115	92,878,236	N/A	55,829,305
No. expressed genes^*b*^		1,868	2,454	N/A	2,235
Accession no.		SRX481527	SRX481475	N/A	SRX481530

a 2004 assembly (Xu *et al.*^2004^).

b Minimum 5X CDS coverage.

All the genomes except *C. hominis* UKH1 were annotated using a semi-automated approach. We trained Augustus (Stanke *et al.*^2004^) using a set of previously manually curated genes. Consensus predictor EVidence Modeler, EVM (Haas *et al.*^2008^), was used to generate annotations based on predictions from Augustus and GeneMark-ES (Borodovsky and Lomsadze 2011), transcripts assembled from RNAseq reads and matches to a set of highly conserved eukaryotic genes—the Core Eukaryotic Genes Mapping Approach genes (Parra, Bradnam and Korf 2007). In addition, 394 genes (∼10% of all genes) in the *C. hominis* TU502_2012 genome were manually annotated using Web Apollo (Lee *et al.*^2013^). The manually curated genes are thought to encode antigens (Ifeonu *et al.*, in preparartion). The *C. hominis* genes TU502_2012 were mapped to the *C. hominis* UKH1 assembly using GMAP (v2015-12-31), and filtered to include only matches that extend at least over 95% of the sequences and have ≥95% alignment identity at the amino acid level. The final assembly attributes are listed in Table 1. This Whole Genome Shotgun project has been deposited in DDBJ/EMBL/GenBank under the accession numbers listed in Table 1 and the sequences are accessible at CryptoDB (http://CryptoDB.org). These are the first versions of genome sequence assemblies and annotations for each isolate.

The genome of *C. hominis* isolate TU502 has been sequenced previously (Xu *et al.*^2004^). We resequenced the genome of this isolate, after multiple passages, in an attempt to improve the reference genome assembly and gene set for this species. The resulting *C. hominis* TU502_2012 genome assembly consists of only 119 contigs, a 10-fold reduction relative to the 2004 assembly. The genome assembly is now more complete, and roughly the same size as that of *C. parvum*, which is also 9.1 Mbp in length (Abrahamsen *et al.*^2004^). The genes in the new annotation are on average 500 bp longer than their counterparts in the original 2004 annotation, resulting in an increase of 17% in the fraction of the genome that encodes for proteins. In order to determine if this gene structural annotation is more accurate than the one published in 2004, we compared the length of all *C. parvum* IOWA II proteins with their orthologs in either *C. hominis* TU502 or *C. hominis* TU502_2012. The distribution of length differences based on the comparison to the 2012 reannotation indeed has lower variance, with an additional 500 genes similar in length between the two species (Fig. 1). Also, there are 538 *C. parvum* genes without orthologs in the *C. hominis* TU502 2004 annotation compared to only 288 such cases in the 2012 annotation. Interestingly, while the original *C. hominis* annotation had a preponderance of genes shorter than their *C. parvum* orthologs, the current gene set is skewed in the opposite direction (Fig. 1). Whether this difference is real, or a result of remaining gene structure errors in one or both species, remains to be determined. The *C. hominis* TU502_2012 annotation contains 206 predicted protein-coding genes with no orthologs in *C. parvum* IOWA II. Of the 3745 predicted protein-coding genes in *C. hominis* TU502_2012, only 63% are also found in all other annotated *Cryptosporidium* genomes available to date: *C. parvum* IOWA II, *C. meleagridis* UKMEL1, *C. baileyi* TAMU-09Q1 and *C. muris* RN66 (Fig. 1). Finally, 110 predicted protein-coding genes are present in the three newly sequenced genomes, but homologs are absent in the current *C. parvum* predicted proteome. These significant differences in gene content among species are, in all likelihood, due mostly to the limitations of the semi-automated annotation approach used, rather than to true instances of gene gain/loss. An intense, manual curation effort of the genome annotation of each species is ongoing, and will be essential to validate these results.

**Figure 1.**
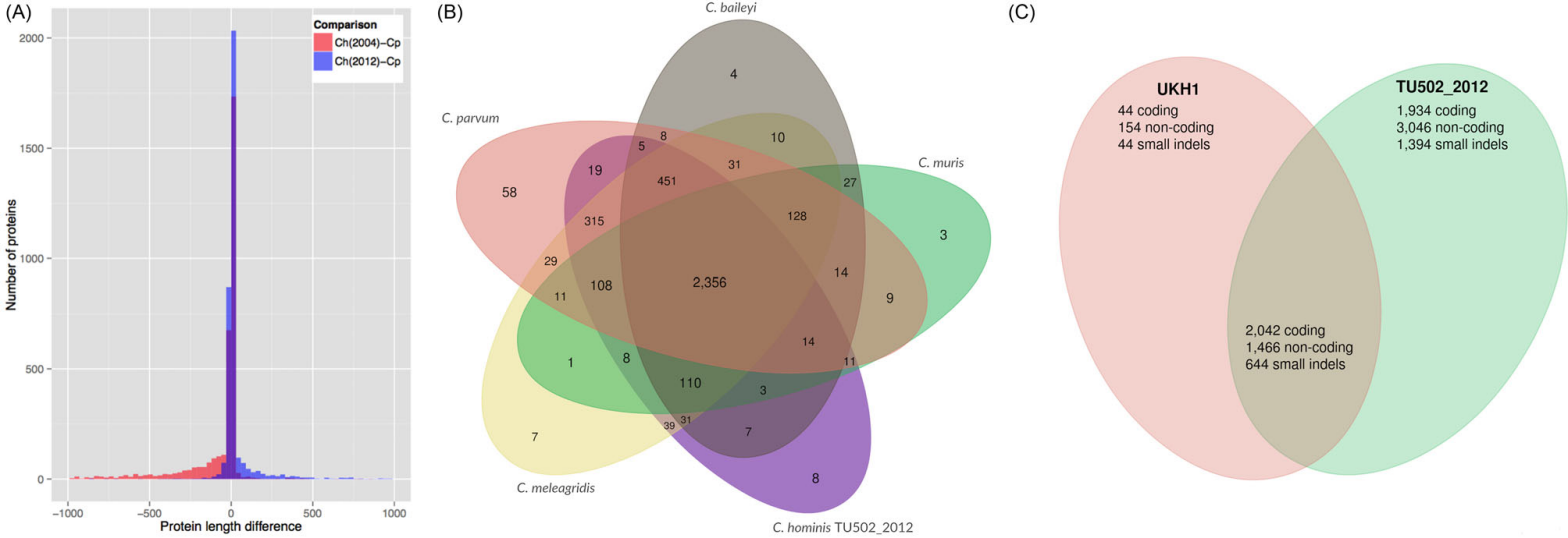
Inter- and intraspecies genome-wide comparisons of genome composition. (**A**) Comparison of protein length between *C parvum* and the 2004 and 2012 versions of the *C. hominis* TU502. (**B**) Distribution of orthologous gene clusters in five *Cryptosporidium* species. (**C**) Distribution of SNPs and short indels among three *C. hominis* isolates, TU502, TU502_2012 and UKH1. DNA sequence reads from the *C. hominis* TU502_2012 and UKH1 were mapped against the reference genome assembly of *C. hominis* TU502, as well as against each other, using BWA (Li and Durbin 2009). SNPs and small indels were identified using GATK (McKenna *et al.*^2010^). Identified variants were further filtered for reliability, according to the following parameter values: (DP < 12) ∥ (QUAL < 50) ∥ (SB > –0.10) ∥ (MQ0 > = 2 && (MQ0/(1.0 * DP)) > 0.1). SNPs were categorized as coding and non-coding, given the assembly and the annotation, using VCFtools.

Genetic differences among *C. hominis* isolates were identified by read mapping, followed by calling and filtering of single nucleotide polymorphisms (SNPs) and small insertions/deletions (indels). A total of 10 526 sequence variants were identified in *C. hominis* TU502_2012 relative to the reference *C. hominis* TU502 assembly; in contrast, only 4394 sequence variants were found between *C. hominis* UKH1 and the reference *C. hominis*. Interestingly, the vast majority of the differences relative to the reference TU502 genome are shared between the two new isolates (Fig. 1). A plausible explanation, which remains to be verified, is that these SNPs common to both new isolates are in fact sequencing errors in the original *C. hominis* TU502 assembly, which was based on low-coverage Sanger sequencing. This, however, does not explain the fact *C. hominis* TU502_2012 has more differences relative to TU502 than does UKH1. It is possible that during the approximate 20 passages in gnotobiotic pigs which *C. hominis* TU502_2012 isolate has experienced between 2004 and 2012, the make-up of the parasite population has shifted. In the absence of methods for cloning and expanding single *Cryptosporidium* sporozoites, the isolates sequenced to date are likely to be heterogeneous populations (Grinberg and Widmer 2016). In fact, high-throughput sequencing of a polymorphic locus demonstrated the presence of multiple alleles in laboratory and natural *Cryptosporidium* isolates (Widmer *et al.*^2015^).

We generated RNAseq data for two of the species, *C. hominis* and *C. baileyi*. These data are strand specific, a tremendous advantage when attempting to generate accurate gene-specific expression values in highly gene-dense genomes, where neighboring transcriptional units often overlap (Tretina, Pelle and Silva 2016). The quantity of RNAseq data generated for *C. hominis* UKH1 was six times than that for the TU502_2012 isolate (Table 1). Despite this difference, the relative expression values for each gene are remarkably similar for the two isolates (*r*^2^ ∼ 0.96; Fig. 2), which supports the strength of the relative expression results. The RNAseq data generated from oocysts indicate that ∼50% and ∼60% of protein-coding genes are expressed in *C. hominis* TU502_2012 and *C. baileyi*, respectively, during this stage of the life cycle (Table 1). Gene expression is also positively correlated between species (*r*^2^ ∼ 0.51; Fig. 2), with lactate/malate dehydrogenase (LDH), a GDP-fucose transporter, agrin and the ubiquitous heat shock protein 90 (HSP90) being among the most highly expressed genes in both species. LDH and HSP90 have been shown to be among the top nine most highly expressed genes in *C. parvum* oocysts (Zhang *et al.*^2012^). Genes preferentially expressed in one or the other species may provide a good starting point to investigate biological differences between taxa. Among the genes that differ most in expression level between the two species are pyridine nucleotide-disulphide oxidoreductase, which has a higher level of expression in *C. hominis*, and AhpC/TSA family protein, WD repeat-containing protein 82 and DNA mismatch repair protein msh-2, all of which have higher expression levels in *C. baileyi*.

**Figure 2.**
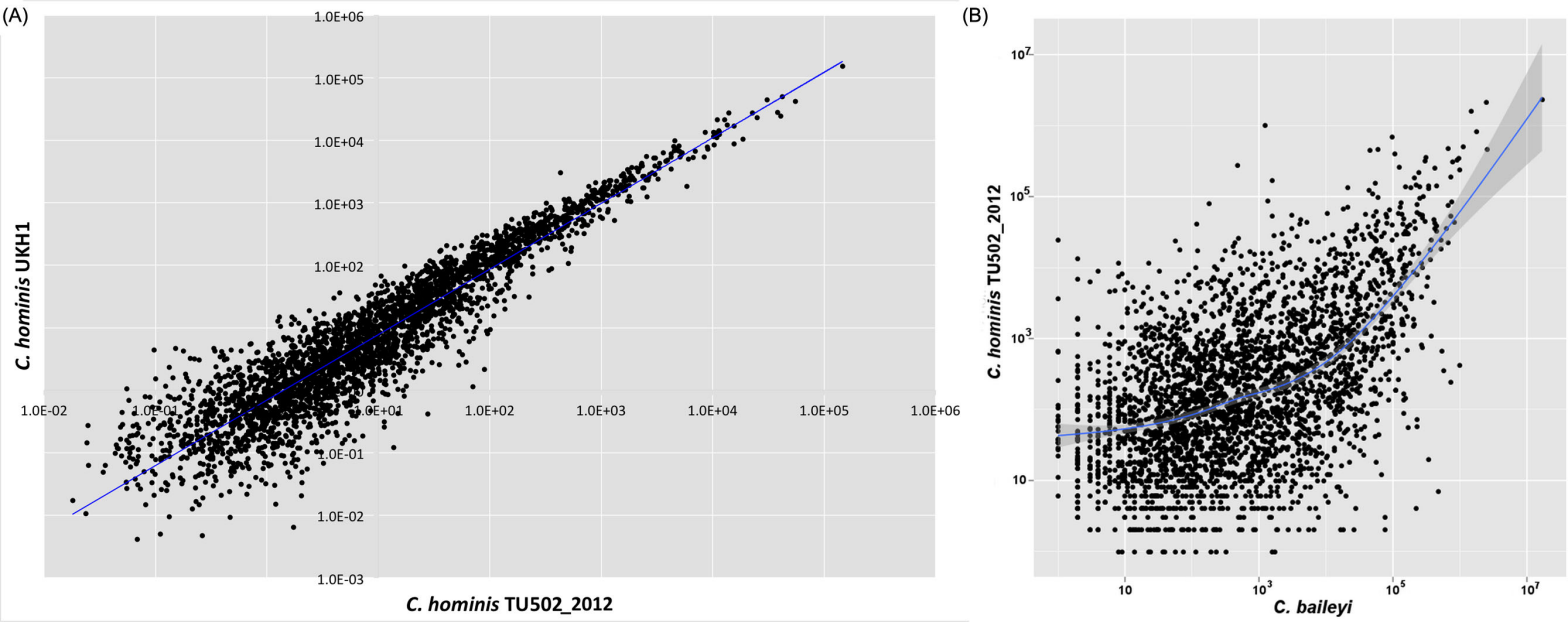
Gene expression in *Cryptosporidium* oocysts is correlated within and between species. (**A**) Correlation in oocyst gene expression is highly correlated between two isolates of *C. hominis* (*r*^2^ ∼ 96%). (**B**) Correlation in oocyst gene expression is correlated between *C. hominis* and *C. baylei* (*r*^2^ ∼ 51%), particularly among the most highly expressed genes.

The work on *Cryptosporidium* genomes and their respective annotations with particular emphasis on the manual curation of the structure and function of all protein-coding genes is continuing. Together with the identification of genes unique to each species and genes with species-specific expression profiles, this work will facilitate the identification of genes responsible for host specificity and other phenotypes relevant to the understanding of cryptosporidiosis.
